# Sequential and Iterative Auto-Segmentation of High-Risk Clinical Target Volume for Radiotherapy of Nasopharyngeal Carcinoma in Planning CT Images

**DOI:** 10.3389/fonc.2020.01134

**Published:** 2020-07-23

**Authors:** Xudong Xue, Nannan Qin, Xiaoyu Hao, Jun Shi, Ailin Wu, Hong An, Hongyan Zhang, Aidong Wu, Yidong Yang

**Affiliations:** ^1^Department of Radiation Oncology, The First Affiliated Hospital of USTC, Division of Life Sciences and Medicine, University of Science and Technology of China, Hefei, China; ^2^School of Biomedical Engineering, Anhui Medical University, Hefei, China; ^3^School of Computer Science and Technology, University of Science and Technology of China, Hefei, China; ^4^School of Physical Sciences, University of Science and Technology of China, Hefei, China

**Keywords:** auto-segmentation, radiotherapy, clinical target volume, deep learning, nasopharyngeal carcinoma

## Abstract

**Background:** Accurate segmentation of tumor targets is critical for maximizing tumor control and minimizing normal tissue toxicity. We proposed a sequential and iterative U-Net (SI-Net) deep learning method to auto-segment the high-risk primary tumor clinical target volume (CTVp1) for treatment planning of nasopharyngeal carcinoma (NPC) radiotherapy.

**Methods:** The SI-Net is a variant of the U-Net architecture. The input of SI-Net includes one CT image, the CTVp1 contour on this image, and the next CT image. The output is the predicted CTVp1 contour on the next CT image. We designed the SI-Net, using the left side to learn the volumetric features and the right to localize the contour on the next image. Two prediction directions, one from inferior to superior (forward direction) and the other from superior to inferior (backward direction), were tested. The performance was compared between the SI-Net and the U-Net using Dice similarity coefficient (DSC), Jaccard index (JI), average surface distance (ASD), and Hausdorff distance (HD) metrics.

**Results:** The DSC and JI values from the forward direction SI-Net model were 5 and 6% higher than those from the U-Net model (0.84 ± 0.04 vs. 0.80 ± 0.05 and 0.74 ± 0.05 vs. 0.69 ± 0.05, *p* < 0.001). The smaller ASD and HD values also indicated a better performance (2.8 ± 1.0 vs. 3.3 ± 1.0 mm and 8.7 ± 2.5 vs. 9.7 ± 2.7 mm, *p* < 0.01) for the SI-Net model. For the backward direction SI-Net model, the DSC and JI values were still better than those from the U-Net model (*p* < 0.01), although there were no significant differences in ASD and HD.

**Conclusions:** The SI-Net model preserved the continuity between adjacent images and thus improved the segmentation accuracy compared with the conventional U-Net model. This model has potential of improving the efficiency and consistence of CTVp1 contouring for NPC patients.

## Introduction

In 2018, about 129,000 people were diagnosed with nasopharyngeal carcinoma (NPC) and about 73,000 people died because of it (1). With the advances of radiation technology, intensity-modulated radiotherapy (IMRT) and volumetric-modulated arc therapy (VMAT) have become standard radiotherapy methods for NPC patients (2). Precise radiotherapy relies on accurate delineation of tumor targets and organs at risk (OARs). In radiotherapy practice, these anatomical structures are usually manually delineated by radiation oncologists on a treatment planning system (TPS). The manual delineation, however, is a time-consuming and labor-intensive process. It usually takes about several hours to contour all structures in NPC radiotherapy planning (3). Moreover, the manual delineation is a subjective process and hence is prone to inter-practitioner variability. The NPC target segmentation is particularly challenging because of the substantial interpatient heterogeneity in tumor shape and the poorly defined tumor-to-normal tissue interface (4), resulting in considerable variations in clinical target volume (CTV) among physicians (5, 6).

Auto-segmentation method has the potential of improving the contouring accuracy and efficiency. Different types of auto-segmentation methods have been reported. Atlas-based segmentation (7–9) is one popular technique. It matches new images to a group of contours selected from a database on the basis of deformable registration. But this method has several disadvantages. For example, it has long computation time and often could not account for large anatomical variations due to the uncertainty of deformable registration (3). In recent years, deep learning has achieved great success in computer science. It has been applied to auto-segmenting tumor targets and OARs in radiotherapy (10–13). Studies have demonstrated that deep leaning method can perform comparably with or even better than manual segmentation for some tumor sites.

In this work, we proposed a sequential and iterative U-Net (SI-Net) model that can automatically segment high-risk primary tumor CTV (CTVp1) in NPC radiotherapy. The SI-Net preserved the continuity between adjacent images and thus improved segmentation accuracy. We trained the model using 135 patients and tested its accuracy using 15 patients. The results showed that the SI-Net performed better than conventional two-dimensional (2D) U-Net did.

## Materials and Methods

### Data

We retrospectively selected 150 NPC patients treated in our hospital between January 2016 and May 2019. The patient demographics are shown in Table 1. The patients with locally advanced cancer (*N* = 53) were treated with induction chemotherapy followed by concurrent chemoradiotherapy, and the remaining patients were treated with either radiotherapy or concurrent chemoradiotherapy. During CT simulation, patients were immobilized in supine position with a thermoplastic mask and underwent contrast-enhanced CT scan on a Somatom Definition AS 40 (Siemens Healthcare, Forchheim, Germany) system. The dimension, resolution, and thickness of CT images were 512 × 512, 0.98, and 2.5 mm, respectively. To better delineate the tumor region, T1-weighted MR images were also acquired and fused with CT images. The CTVp1 was delineated by experienced radiation oncologists on the CT images in a Pinnacle TPS (Philips Radiation Oncology System, Fitchburg, WI, USA) following the international guideline for NPC CTVp1 delineation (5).

**Table 1:** Demographics of enrolled NPC patients.

**GENDER**
Male	112 (74.7%)
Female	38 (25.3%)
**AGE**
Median	52.0
Range	22.0–80.0
**T STAGE**
T1	11 (7.3%)
T2	23 (15.3%)
T3	79 (52.7%)
T4	37 (24.7%)
**N STAGE**
N0	8 (5.3%)
N1	41 (27.3%)
N2	84 (56.0%)
N3	17 (11.3%)
**TREATMENT OPTIONS**
Induction chemotherapy + concurrent chemoradiotherapy	53 (35.3%)
Radiotherapy or concurrent chemoradiotherapy	97 (64.7%)
**CTVp1 VOLUME (ml)**
Range	173–741
Mean	264 ± 80

*NPC, nasopharyngeal carcinoma; CTVp1, primary tumor clinical target volume*.

### Image Preprocessing

A binary body mask was automatically created in each CT image to separate the body from external structures, such as the couch, immobilization plate, and thermoplastic mask. First, the Otsu thresholding was applied to each CT image. Then the body mask was generated after the gaps and holes in the image were filled with morphological closing operation. Subsequently, multiplication of the CT image and the body mask produced the final image used in the deep learning analysis. Images were flipped and random rotated to augment the training dataset.

### Network Architecture

**Figure 1:**
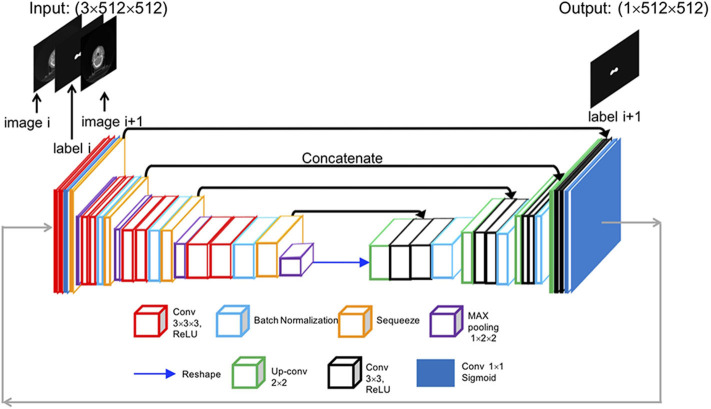
Overall architecture of the proposed SI-Net model. SI-Net, sequential, and iterative U-Net.

The SI-Net is a variant of U-Net (14), which is popular convolutional network architecture for biomedical image segmentation. The U-Net consists of a contracting path to capture context through convolution and max-pooling operations and a symmetric expanding path to localize features through up-convolution and concatenation operations. The U-Net architecture enables structure delineation on one isolated image. It, however, does not consider the continuity between neighboring images in a three-dimensional (3D) image environment. We modified the U-Net architecture and designed the SI-Net to specially take the image continuity into account. The architecture of the SI-Net is illustrated in Figure 1. The input is three 512 × 512 matrices, including the current CT image, the CTVp1 contour on the current image, and the next adjacent image. The output is the CTVp1 contour on the next adjacent image, which is also one of the two input images. A manual CTVp1 contour is required on the beginning image as the input. The predicted contour will work as the input for subsequent images. The left side of the architecture consists of 3D convolutions to learn the volumetric features, and the right side consists of 2D operations to localize the contour on the next image. In the left, each layer contains two 3 × 3 × 3 convolutions each followed by a rectified linear unit (ReLU) activation (15) and one 2 × 2 × 2 max pooling with two strides in each dimension. To better concatenate the 3D convolutions on the left side with the 2D convolutions on the right side, the 3D convolution is down-sampled by a 3 × 1 × 1 max pooling and then squeezed to decrease channels. A reshape layer is used at the bottom of the architecture. On the right side, each layer consists of three processes: one 2 × 2 convolution for up-sampling, one concatenation with the corresponding feature map from the left side, and two 3 × 3 convolutions to recover object segmentation details. In the last process, each convolution was followed by a ReLU activation. The final layer is a 1 × 1 convolution activated by a sigmoid function. All ReLU activations were followed by batch normalization (16).

### Training Process

Of the total 150 patients, 120 were chosen as the training set, 15 patients as the validation set, and the remaining 15 patients as the testing set. The manual contours were taken as the ground truth. The loss function used in the study was 1—DSC index. The Nesterov Adam optimizer was used with a learning rate of 0.0001. The network architecture was implemented in Python using the Keras package (17) on a Supermicro workstation with an Intel Xeon Processor E5-2695 CPU and an NVIDIA Tesla P100 GPU. Two predicting directions, one from inferior to superior (forward direction) and the other from superior to inferior (backward direction), were tested. The results were compared with those from U-Net model.

### Evaluation Metrics

The performance of the SI-Net auto-segmentation algorithm was evaluated with Dice similarity coefficient (DSC), Jaccard index (JI), average surface distance (ASD), and Hausdorff distance (HD). The definitions of these metrics are described in Table 2.

**Table 2:** The definitions of evaluation metrics.

**Metrics**	**Mathematical definition**	**Meaning**	**Range**
Dice similarity coefficient (DSC)	$DSC\left(A,B\right)=\frac{2|A\cap B|}{\left|A|+|B\right|}$	Overlap between the two contours	0–1
Jaccard index (JI)	$JI\left(A,B\right)=\frac{\left|A\cap B\right|}{\left|A\cup B\right|}$	Similarity of the two contours	0–1
Average surface distance (ASD)	$ASD=\frac{{\vec{d}}_{H,avg}\left(A,B\right)+{\vec{d}}_{H,avg}\left(B,A\right)}{2}$ ${\vec{d}}_{H,avg}\left(A,B\right)=\frac{1}{\left|A\right|}\sum_{a\in |A|}\min_{b\in |B|}d\left(a,b\;\right)$	Average surface distance between two contours (mm)	≥0
Hausdorff distance (HD)	*H*(*A, B*) = max(*h*(*A, B*), *h*(*B, A*)) $h\left(A,B\right)=\underset{a\in A}{\max}\left(\underset{b\in B}{\min}||a-b||\;\right)$	Maximum surface distance between two contours (mm)	≥0

### Statistical Analysis

The paired *t*-test was performed to compare the DSC, JI, ASD, and HD values between different models. The data were presented with mean ± standard deviation. The significance was determined at *p* < 0.05. All analyses were performed using SPSS version 16.0 software.

## Results

**Figure 2:**
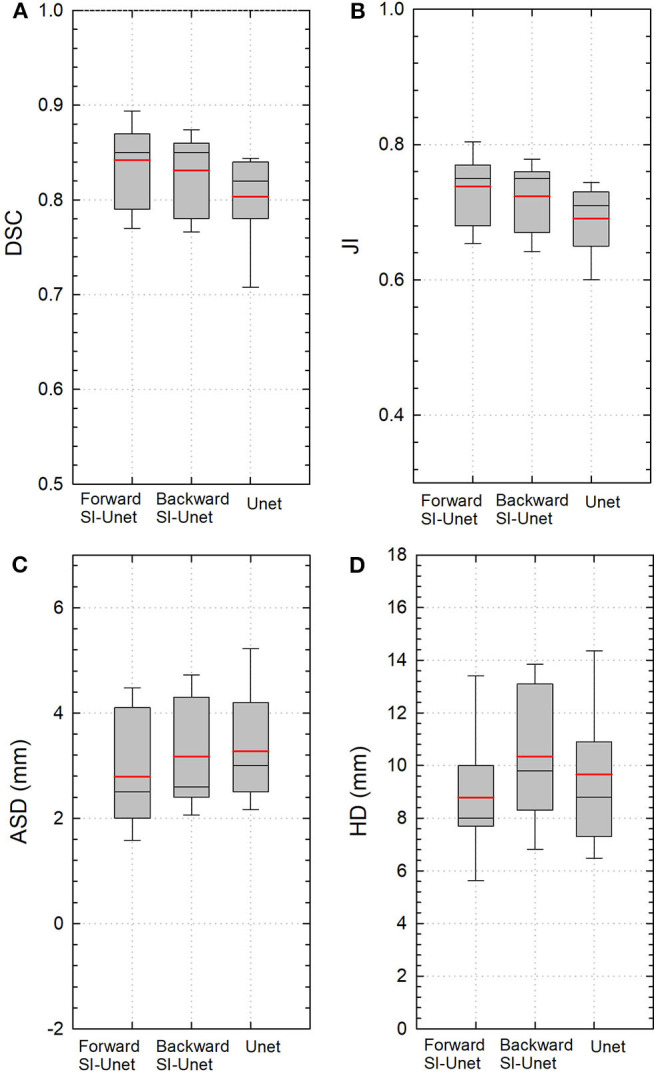
Boxplots of **(A)** DSC, **(B)** JI, **(C)** ASD, and **(D)** HD values from three different models. Red and black lines represent the mean and median values, respectively. The boxes indicate the 25th and 75th percentiles; the error bars indicate the 10th and 90th percentiles. DSC, Dice similarity coefficient; JI, Jaccard index; ASD, average surface distance; HD, Hausdorff distance.

The performance of the proposed SI-Net for all 15 test patients is shown in Figure 2. The average DSC and JI values from the SI-Net were 5% and 6% higher than those from the U-Net (0.84 ± 0.04 vs. 0.80 ± 0.06, *p* < 0.001; 0.74 ± 0.05 vs. 0.69 ± 0.05, *p* < 0.001), indicating that the SI-Net performed better than the U-Net did. The smaller ASD and HD values further confirmed the advantage of the SI-Net over the U-Net (2.8 ± 1.0 vs. 3.3 ± 1.0 mm, *p* = 0.006; 8.7 ± 2.5 vs. 9.7 ± 2.7 mm, *p* = 0.008).

The performance of the SI-Net using backward prediction direction is also shown in Figure 2. The DSC and JI values were still better than those from the U-Net (0.83 ± 0.04 vs. 0.80 ± 0.05, *p* = 0.008; 0.72 ± 0.05 vs. 0.69 ± 0.05, *p* = 0.004), although the differences in ASD and HD were not significant (3.1 ± 1.0 vs. 3.3 ± 1.0 mm, *p* = 0.616; 10.3 ± 2.6 vs. 9.7 ± 2.7 mm, *p* = 0.223).

**Figure 3:**
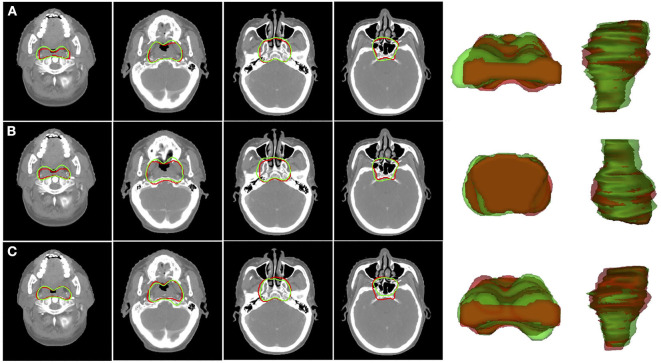
Segmentation results for CTVp1 with different CNN model: **(A)** SI-Net with forward direction; **(B)** SI-Net with backward direction; and **(C)** U-Net model. Red lines denote the manual ground truth contours, and green lines represent auto-segmentation results. CTVp1, primary tumor clinical target volume; CNN, convolutional neural network; SI-Net, sequential and iterative U-Net.

Figure 3 shows the 2D and 3D visualizations of the auto-segmented contours for one patient. Red lines represent manual contours, and green lines auto-segmented ones. Generally, the auto-segmentation was close to the manual segmentation, which was the ground truth (Figure 3A). Figure 3B presents the auto-segmented contours predicted with the backward direction, which were slightly different from those predicted with forward direction. Figure 3C presents the segmentation results from the U-Net. Overall, the SI-Net preserved the connection between adjacent images and better maintained the continuity of the adjacent contours.

The time needed to train the SI-Net and U-Net for 200 epochs was 12 and 8 h, respectively. The mean time for CTVp1 auto-segmentation was 20 and 13 s per patient, respectively, which were much less than the manual contouring time (typically 10–20 min per patient).

## Discussion

**Figure 4:**
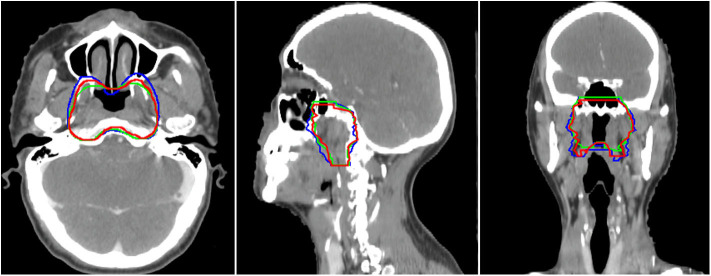
The three manual CTVp1 contours by different physicians for one patient. CTVp1, primary tumor clinical target volume.

In this study, we proposed a novel SI-Net neural network to auto-segment the CTVp1 for NPC patients. The SI-Net performed significantly better than U-Net did. In addition, to benchmark the SI-Net against manual contours, we conducted an independent and separate pilot study. In the pilot study, three patients were randomly selected, and their CTVp1 was re-contoured by three radiation oncologists, each with more than 6-years experiences in head and neck cancer radiotherapy. The manual contours were then cross-compared among the three physicians to obtain the inter-practitioner variability. The evaluation metrics are shown in Table 3. The DSC values range from 0.84 to 0.90, JI from 0.74 to 0.82, ASD from 1.69 to 2.74 mm, and HD from 4.76 to 6.98 mm. These values serve as references for the auto-segmentation. The manual contours are also demonstrated in Figure 4. The SI-Net was able to achieve a contouring accuracy comparable with that by radiation oncologists. In Figure 4, which demonstrates all three manual contours for one patient, it can be observed that most disagreements between physicians in CTVp1 contouring took place in the anterior and inferior borders, which lack soft tissue contrast.

**Table 3:** Comparison of the manual contours by three different oncologists.

	**DSC**	**JI**	**ASD (mm)**	**HD (mm)**
Oncologist 1 vs. 2	0.88 ± 0.05	0.80 ± 0.07	1.88 ± 0.43	5.48 ± 1.54
Oncologist 2 vs. 3	0.84 ± 0.07	0.74 ± 0.11	2.74 ± 1.04	6.98 ± 1.80
Oncologist 3 vs. 1	0.89 ± 0.05	0.81 ± 0.07	1.79 ± 0.66	4.86 ± 1.42

*DSC, Dice similarity coefficient; JI, Jaccard index; ASD, average surface distance; HD, Hausdorff distance*.

Accurate segmentation of the tumor target is critical to maximizing tumor control and minimizing radiation toxicities. The CTVp1 in NPC radiotherapy includes both the tumor's gross tumor volume (GTV) and the nearby volumes that may harbor subclinical and microscopic cancer spread. The lack of soft tissue contrast on CT images and hence poorly defined tumor-to-normal tissue interface makes the CTVp1 delineation a challenging task, especially for junior physicians. Considerable variability exists in manual contouring even for experienced radiation oncologists, which was observed in our separate pilot study. From this point of view, deep learning-based auto-segmentation can play a role. It has potential of improving the contouring consistency and accuracy through learning from a large set of contours manually contoured by experienced radiation oncologists. In addition, the time spent on CTVp1 contouring varies between senior and junior physicians. The deep learning auto-segmentation method can provide, at least, a good start point from which they can improve and finalize the contours. Therefore, there are more and more interests in applying deep learning methods to auto-contouring in radiotherapy. For instance, Ibragimov and Xing used convolutional neural networks (CNNs) to segment head and neck OARs on CT images, and they showed DSC values from 37.4 to 89.5% (18). Zhu et al. proposed an end-to-end atlas-free deep learning model and demonstrated an average DSC of 78.8% (19). Sun et al. developed a locating-and-segmentation approach and achieved DSC values of 82.2–94% (20). For GTV segmentation, Lin et al. developed a 3D CNN method to auto-contour GTV in MR images, and they demonstrated a DSC value of 0.79 (19). Ma et al. combined a CNN model and a 3D graph cut-base method, and they achieved a DSC value of 85.1% (3).

The SI-Net model we proposed was able to maintain the continuity of contours between adjacent images. The input requirement of the contour on the beginning image is to assist the algorithm to decide the starting location along the superior-to-inferior direction. In spite, it does not rely on contouring directions. Physicians are free to choose their favorite contouring direction when using the SI-Net method. Nonetheless, this is still a feasibility study and warrants follow-up studies before the proposed method can be translated into clinic use. On the other hand, we only performed CTVp1 segmentation. In the future, we will test this hypothesis of using the SI-Net to auto-segment nodal CTV.

Although our method has achieved decent segmentation accuracy, there are still several limitations. First, the total training and validation datasets have only 135 patients, which is relatively a small number. Increasing the training dataset could further improve the accuracy and robustness. Second, the inter-practitioner variability on CTVp1 delineation in the training dataset may compromise the training process, although all the radiation oncologists followed a same guideline. Third, MR images were used when the physicians manually contour the CTVp1 but was not included in the auto-segmentation process. We may be able to further improve the segmentation by including MR images into the input of the SI-Net, considering their superior soft tissue imaging contrast.

## Conclusion

In this work, we proposed a novel SI-Net based deep learning method to auto-segment the high-risk primary tumor CTVp1 on NPC radiotherapy patients. The SI-Net preserved the continuity between adjacent images and thus improved the segmentation accuracy when compared with the conventional U-Net. This model has potential of improving the efficiency and consistency of the CTVp1 contouring in the treatment planning of head and neck radiotherapy.

## Data Availability Statement

The datasets generated for this study are available on request to the corresponding author.

## Ethics Statement

This study was carried out in accordance with the Declaration of Helsinki and approved by the Ethics Committee of the First Affiliated Hospital of USTC with reference number: 2020-P-002.

## Author Contributions

XX and NQ selected the enrolled patients and performed the code and data analysis. XH and JS helped with the coding problem. HZ and AidW checked the target delineation. HA and AilW gave useful discussions. YY and XX designed the study and wrote the manuscript. All authors contributed to the article and approved the submitted version.

## Conflict of Interest

The authors declare that the research was conducted in the absence of any commercial or financial relationships that could be construed as a potential conflict of interest.
